# Study on Construction of a Medical X-Ray Direct Digital Radiography System and Hybrid Preprocessing Methods

**DOI:** 10.1155/2014/495729

**Published:** 2014-06-11

**Authors:** Yong Ren, Sheng Wu, Mijian Wang, Zhongjie Cen

**Affiliations:** College of Communication Engineering, Chongqing University, Chongqing 400044, China

## Abstract

We construct a medical X-ray direct digital radiography (DDR) system based on a CCD (charge-coupled devices) camera. For the original images captured from X-ray exposure, computer first executes image flat-field correction and image gamma correction, and then carries out image contrast enhancement. A hybrid image contrast enhancement algorithm which is based on sharp frequency localization-contourlet transform (SFL-CT) and contrast limited adaptive histogram equalization (CLAHE), is proposed and verified by the clinical DDR images. Experimental results show that, for the medical X-ray DDR images, the proposed comprehensive preprocessing algorithm can not only greatly enhance the contrast and detail information, but also improve the resolution capability of DDR system.

## 1. Introduction

Direct digital radiography (DDR), also known as DR, is a direct digital X-ray imaging technology. DR is developed just in recent years as a new digital imaging technology; currently, in the industry, DR system is designed for the X-ray imaging systems using flat-panel detector. The flat-panel detector was successfully developed and applied in clinical domains, which is a leap forward in imaging technology, and flat-panel digital detector replaces the conventional X-ray imaging chain consisting of image intensifier, camera, optical system, and analog to digital converter, resulting in forming the so-called direct digital systems. So it can avoid the influence of image generation exerted by the many aspects of imaging chain, reduce the image noise and distortion, improve the image contrast and resolution, and extend the dynamic range of image by adjusting the window width and position. Another important aspect is the image size increasing (such as 17′′ × 17′′,  17′′ × 14′′), bringing greater vision for clinical application.

The core technology of DR is adopted as X-ray detector plate and computer for image capturing and processing; according to different detection methods, currently, DR technology can be mainly classified into two kinds: the DR equipment based on amorphous silicon or amorphous selenium-based flat-panel detector and the DR equipment based on cesium iodide (CsI) phosphor plate + CCD (charge-coupled devices) imaging technology. The latter technology is introduced by Philips, IDC Canada, Switzerland Rays, and so forth, which is the rise of DR technology in recent years. Its outstanding feature is that the purchase and maintenance costs are significantly reduced under the premise of ensuring the image quality (16 million pixels), and it does not require correction in the period of use; the service life of CsI film panel is up to 4 years; even after the use period, we only need to replace the CsI film panel with other parts still continuing to be used. The contained CCD camera has the characteristics of high resolution, high reliability, high stability, and low cost.

This paper proposes a medical X-ray DR system based on CsI phosphor plate and CCD imaging sensor, referred to as CCD-DR. Meanwhile, for the situation in which output original image from CCD-DR system cannot yet be directly observed and used, it needs to execute a specific image preprocessing work and to particularly study the image enhancement processing technology [1]. On the basis of building CCD-DR system, this paper designs a set of pretreatment methods including image correction and contrast enhancement processing, which can be directly used to observe and analyze by the doctor.

## 2. CCD-DR System Construction

**Figure 1:**
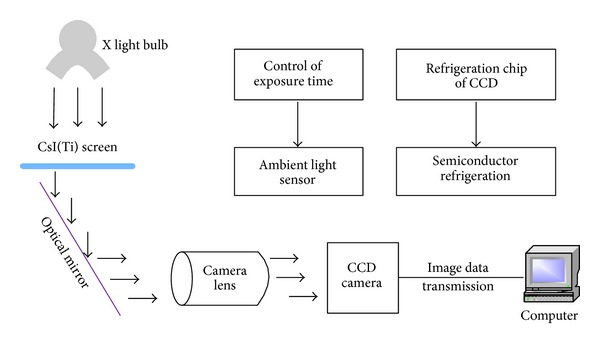
Schematic diagram of the proposed medical X-ray CCD-DR imaging system.

The composition principle of the medical X-ray machine CCD-DR imaging system constructed in this paper is shown in Figure 1.

In this system, carbon fiber substrate CsI 17′′ × 17′′ fluorescent plate from Hamamatsu Company has been used to receive the X-ray irradiation and then produce the visible light image. The CCD camera unit and the optical part are designed separately. The image transmission uses gigabit network interface, and the X-ray machine exposure is controlled by RS-232 serial communication, which needs to detect X-ray exposure intensity and inform the CCD camera for exposure. Terminal computer takes charge of system control, image acquisition, and processing analysis, which are reflected in a comprehensive set of DR imaging workstation software.

### 2.1. CCD Camera Unit Design

The large-area photosensitive KFC-16803 CCD chip (Kodak Company Inc.), which was mainly used in aviation and aerospace due to its high resolution and high performance in SNR and low-noise level, was introduced into our medical DR system. The basic parameters of CCD camera are shown in Table 1.

In order to further decrease the background noise, a refrigeration unit was used in our CCD camera, in which semiconductor refrigeration tablets, temperature sensors, and the control circuit are coupled together to cool the CCD camera to minus 10°C; simultaneously, the craft of preventing condensation on CCD surface should be taken into consideration.

### 2.2. Optical Unit and Mechanical Design

**Figure 2:**
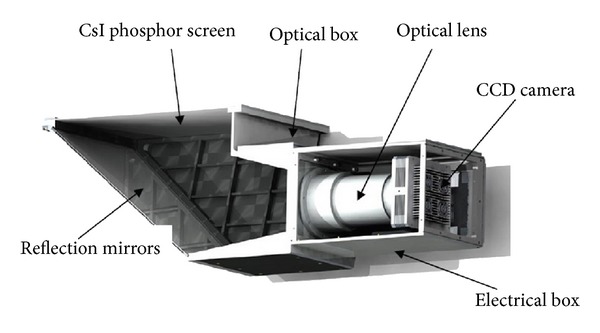
Optical unit.

Figure 2 shows an illustration of optical unit which includes CCD camera, optical lens, optical box, electrical box, reflection mirrors, and CsI phosphor screen.

Reflection mirrors are used for reflecting incident visible light from CsI screen to CCD camera in order to fold and reduce optical path. The mirrors should meet the following requirements:

- glasses with large size, low coefficient of expansion, and good flatness (up to *λ*/2);
- specialized optical reflection coated film with high reflection coefficient (up to 96%);
- hard protective coating layer (aluminized) to prevent the deformation of mechanical structure;
- 17′′ × 17′′ fluorescent plate being trapezoidal with the long side 450 mm, short side 300 mm, height 495 mm, and thickness 8 mm.

Optical lens is used for optical converting from images to CCD target surface. The best object distance of common imaging lens is generally more than 2 meters, and the largest object distance of the DDR system cannot be more than 1 meter; there must be a target specifically designed so as to achieve the best results. In order to reduce the overall size of the volume of the DR system, the desired object distance ranges from 600 to 650 mm. In addition, it must meet the demands of high resolution, large relative aperture, small distortion, no vignetting, and high transfer function. System parameters are listed as follows:

 object distance: 600 mm;
 focal length: 55.26 mm;
 diameter of the imaging plane: 52 mm;
 back working distance: 11.1 mm;
 the optical diameter: 110 mm;
 relative aperture: 1 : 0.92;
 best forward working distance: 600 mm;
 focus mode: mannual;
 wavelength range: 430 nm–702 nm.

### 2.3. Calculation of the Limited Line-Pair Resolution of DR Imaging

We can calculate the limited line-pair resolution of DR imaging system in the following way.

Here, the size of detecting fluorescence plate is (17′′ × 17′′) conversed into the horizontal size with mm is 17 × 25.4 mm = 431.8 mm. Simultaneously, CCD camera pixels' number is 4096 × 4096 (16 million).

So, when performing the CCD imaging, each pixel's corresponding flat target size on horizontal direction is 431.8 mm/4096 = 0.1054 mm; then, the size of each line pair (i.e., 2 pixels with one being white and one being black) is the reciprocal answer of 2 × 0.1054 mm ≈ 0.211 mm; that is, 1/0.211 mm = 4.7 LP/mm.

The text above about LP quota is theoretical, and the ultimate resolution of the CCD chip is well matched, but, practically, with the influence of other links, it is impossible to absolutely reach this level of quota. Also, it can come to a conclusion that the crucial factor to determine the DR imaging LP is the effective pixels' number of the CCD camera.

## 3. Comprehensive Preprocessing Method 

**Figure 3:**
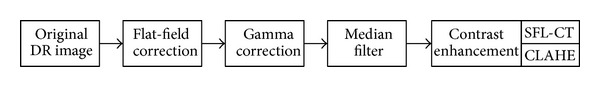
Flowchart of the proposed comprehensive preprocessing method.

Before the DR image can be used for clinical analysis, the original DR images without any correction will be processed serially by computer. The flowchart of the proposed comprehensive preprocessing method is demonstrated in Figure 3. Original DR image → image flat-field correction → image gamma correction → median filter → image contrast enhancement which consists of two basic modes: sharp frequency localization-contourlet transform (SFL-CT) and contrast limited adaptive histogram equalization (CLAHE).

### 3.1. Flat-Field Correction

Indeed, CCD cameras vary in response to photosensitive element, the noise level, the quantum efficiency, and so forth; under the same conditions, each photosensitive element will produce different photoelectrons, and the corresponding output signal is inconsistent, which is known as the photo response nonuniformity. For the array of detectors, the phenomenon of photo response nonuniformity is always existing, and CCD-DR system uses a lens and other optical coupling devices, so that much more serious photo response nonuniformities of the system are shown. Especially because of the lens and the X-ray machine tube, bigger differences of the brightness exist at the center of and around the captured image. Flat-field correction can effectively eliminate these differences, making the output image fully reflect the actual captured image. In this paper, we use a simple flat-field correction method.

First, an original image for correction needs to be obtained without irradiation target under the following three exposure conditions. (1) The first condition is having a fully black image. Adjust the dose of X-ray machine to the minimum; close collimator completely, and capture 10 images continuously; at last, average them to get the fully black image. (2)  The second condition is having a 40% brightness image. Adjust the dose of X-ray machine to make the brightness of the central area of the captured image about 0x6666 = 0xFFFF∗40%; at this dose, continuously capture 10 images, and average them to obtain 40% brightness image. (3) The third condition is having a 60% brightness image. Adjust the dose of X-ray machine, so that the brightness of the central area of the captured image is about 0x9999 = 0xFFFF∗60%; at this dose, continuously capture 10 images, and average them to obtain the 60% brightness image.

Then, add the 40% brightness image to the 60% brightness image to get a white image, and we use the above obtained fully black image. Store the fully white image and the fully black image into the system's hard drive, as they are used as the reference images after every image capturing.

The flat-field correction formula is as follows:

(1) $$\begin{array}{c} a\left[x,y\right]=\left\{\frac{c\left[x,y\right]-\text{BLACK}\left[x,y\right]}{\text{WHITE}\left[x,y\right]-\text{BLACK}\left[x,y\right]}\right\}\times 0\text{xFFFF}. \end{array}$$

Here, *a*[*x*, *y*] is the pixel value after correction, *c*[*x*, *y*] is the pixel value of current image, BLACK [*x*, *y*] is the pixel value of fully black image, and WHITE [*x*, *y*] is the pixel value of fully white image.

As the (WHITE [*x*, *y*] − BLACK [*x*, *y*]) in the denominator, this value cannot be zero; in fact, if the value of (WHITE [*x*, *y*] − BLACK [*x*, *y*]) is close to 0 or below a certain threshold, it can be considered that this point is dead pixel of CCD, which should be replaced with field value.

After the X-ray exposure, all the collected original images are initially processed by the flat-field correction algorithm, resulting in achieving consistent and homogeneous DR images.

### 3.2. Gamma Correction

The image generated from medical X-ray machine generally requires gamma correction processing, so as to make it meet the display characteristics and to be easy to the human eye for observation. And the correction factor should be set to be adjusted manually. The principle is as follows:

(2) $$\begin{array}{c} a\left[x,y\right]={c[x,y]}^{1/\gamma }. \end{array}$$

Here, *a*[*x*, *y*] is the pixel value of correction; *c*[*x*, *y*] is the pixel value of current image, and *γ* is the correction factor.

### 3.3. Hybrid Image Contrast Enhancement Algorithm

Theory and practice in the past have proved that multiscale medical image contrast enhancement approaches, such as MUSICA algorithm [2] and wavelet-based methods [3], are effective for improving X-ray imaging quality. In order to get better image, a hybrid image contrast enhancement algorithm followed by flat-field and gamma corrections is proposed. This algorithm consists of three parts: a median filter (5 × 5 window) aiming at eliminating salt and pepper noise, SFL-CT-based enhancement algorithm, and CLAHE-based enhancement algorithm.

#### 3.3.1. SFL-CT-Based Enhancement Algorithm

**Figure 4:**
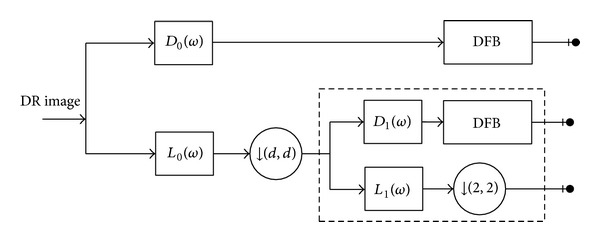
Block diagram of SFL-CT for 1 level decomposition.

DR images are always characterized by low contrast and overexposure, which makes them hard to preprocess, such as image denoising and contrast enhancement. We apply the SFL-CT-based enhancing algorithm to implement image edge enhancement. As we know, contourlet transform (CT) [4] has good performance in representing the image salient features such as lines, edges, curves, and contours because of its anisotropy and directionality. The SFL-CT [5] is one improvement of the CT. It replaces the Laplacian pyramid transform in the old version with sharp frequency localization pyramidal filter banks (SFL-FB) to achieve multiscale decomposition for the image. Thus, it has much better localization in the frequency domain and regularity in the spatial domain compared with the old version, and it can significantly inhibit the spectrum aliasing. The block diagram of SFL-CT is shown in Figure 4.

As shown in Figure 4, the SFL-CT is constructed as a combination of the SFL-FB and the directional filter banks (DFB). Conceptually, first, the SFL-FB captures point discontinuities; then, directional filter banks follow it to link point discontinuity into linear structure. In SFL-FB, *L*_0_(*ω*), *D*_0_(*ω*) are united as filter bank FB1, and *L*_1_(*ω*), *D*_1_(*ω*) are united as filter bank FB2. We use *L*_0_(*ω*), *L*_1_(*ω*) to represent the low-pass filters and *D*_0_(*ω*), *D*_1_(*ω*) to represent the high-pass filters in the multiscale decomposition.

The proposed enhancing algorithm [6, 7] can be depicted as the following flow pattern: (1) process the image with SFL-CT; (2) manipulate the SFL-CT coefficients; (3) reconstruct the modified coefficients of SFL-CT to get the enhanced image.

We introduce the noise deviation *σ*  in the SFL-CT domain. Considering the fact that the magnitudes of noise coefficients at different scales are all very small, we believe that the coefficients whose magnitude is less than *ασ* are the noise, and they are not to be enhanced. We think of the coefficients whose magnitude is greater than *ασ* and less than *t* as the weak edges, which need the corresponding enhancement, and the enhanced extent is controlled by *t* and *q*. And we divide the weak edges into two ranges by 2*ασ*, and their coefficients can be calculated by two different equations as displayed in the following. The coefficients whose magnitude is greater than *t* can be considered as the strong edges, which should be attenuated to some extent. Enhancing the weak edges as well as attenuating the strong edges to some extent can further highlight the enhancement effect of weak edges. Based on the above description, we give the following nonlinear enhancement function.

Consider

(3) $$\begin{array}{c} y_{\alpha }\left(x,\sigma \right)=\{\begin{array}{cc} 1 & \text{if}\;x<\alpha \sigma \\ \frac{x-\alpha \sigma \;}{\alpha \sigma }{\left(\frac{t}{\alpha \sigma }\right)}^{q}+\frac{2\alpha \sigma -x\;}{\alpha \sigma } & \text{if}\;\alpha \sigma \leq x<2\alpha \sigma \\ {\left(\frac{t}{x}\right)}^{q} & \text{if}\;2\alpha \sigma \leq x<t\\ {\left(\frac{t}{x}\right)}^{s} & x\geq t. \end{array} \end{array}$$

Here, *x* indicates the magnitude of SFL-CT coefficients, *σ* denotes the standard noise deviation, *y*_*α*_(*x*, *σ*) represents the nonlinear enhancement function, *t* determines the degree of nonlinearity, and *s* describes the dynamic range compression. Using a nonzero *s* will enhance the faint edges and soften the strong edges. *q* can control the enhanced extent. *α* is a normalization parameter. The *t* parameter is the value under which coefficients are amplified; obviously, this value depends on the coefficients in different scales; *t* can be derived as in the following two options.

- *t* = *F*_*t*_ *σ*, where *F*_*t*_ is independent of the SFL-CT coefficient values.
- *t* = *lM*_*α*_, where *l* is an adjustment parameter, which should be less than 1. *M*_*α*_ is the maximum SFL-CT coefficient within a specific directional subband.

Above all, the specific steps can be summarized as follows.

(1) Use the robust median estimator to estimate the noise standard deviation *σ* in the input image as follows:
  (4) $$\begin{array}{c} \sigma^{2}=\frac{\text{Median}\left(\left|\omega_{i}\right|\right)}{0.6475\;}\;\omega_{i}\in HH, \end{array}$$
  where *HH* describes the finest diagonal band after one level decomposition.
(2) Process the input image with SFL-CT, and calculate the noise standard deviation *σ*_*j*,*k*_ of every directional subband *V*_*j*,*k*_  (*j* < *L*,  *K* < *l*_*j*_), where *L* indicates the total decomposition levels and *l*_*j*_ indicates the directional subband numbers of the *j*th decomposition.
(3) For each directional subband *V*_*j*,*k*_,
  (a) calculate the maximum value *M*_*j*_ of the SFL-CT coefficients in this subband, so as to determine the *α*, *t*, and so forth;
  (b) multiply each SFL-CT coefficient *C*_*j*,*k*_  by *y*_*α*_(*C*_*j*,*k*_, *σ*_*j*,*k*_).
(4) Reconstruct the modified coefficients of SFL-CT to get the enhanced image.

#### 3.3.2. CLAHE-Based Enhancement Algorithm

This paper applies the contrast limited adaptive histogram equalization (CLAHE) algorithm, which is developed from the basis of local area histogram equalization (LAHE) algorithm, in order to further improve the local contrast.

Under the condition of being fixed in the related area, in the LAHE algorithm, the local histogram of a pixel (*x*, *y*) of the image is equal to the local histogram of a rectangular window with this pixel as its center. This construction method of local histogram only considers pixels within a local area while ignoring pixels from other areas of image. According to visual characteristics of human, on the one hand, the visual system changed with the change of related areas; on the other hand, it is also affected by the surrounding environment of related areas. Cromartie and Pizer [8] and Louis et al. [9] had proposed a constrained local histogram construction method; this constrained local histogram takes both inner window and outer window histograms into consideration, which means that it consists of two parts: histogram within the rectangular window and histogram outside the rectangular window.

Consider

(5) $$\begin{array}{c} h_{L}\left(r\right)=\alpha h_{W}\left(r\right)+\left(1-\alpha \right)h_{B}\left(r\right), \end{array}$$

where *h*_*W*_(*r*) is normalized histogram within the window, *h*_*B*_(*r*) is normalized histogram outside of the window, and 0 ≤ *α* ≤ 1. Assume that *A*_*W*_ and *A*_*B*_, respectively, represent the areas of the region *W* and the region *B*. If *α* = *A*_*W*_/(*A*_*W*_ + *A*_*B*_), then *h*_*L*_(*r*) = *h*(*r*); this means that the local histogram is equal to the global histogram. If *α* > *A*_*W*_/(*A*_*W*_ + *A*_*B*_), then the local histogram emphasizes local information. In this case, the impact on the relevant area from surrounding environment can be simulated by regulating the local histogram with *α*. Applying this method to construct local histogram, all the local histograms are the same gray level as the global histogram, while each gray level of them has different amplitudes at different locations.

Considering the fact that the impact of pixels far from sliding window on its center pixel is small, we take a little correction on the above histogram construction method. Select a neighborhood of a certain pixel (*x*, *y*) in the image, which consists of the neighborhood of center *W*_1_ and the neighborhood of background *W*_2_. If *m* = 1, then the neighborhood of center *W*_2_ consists of the center pixel (*x*, *y*) and the neighborhood of background consists of the other 8 pixels except the center pixel. For a given *m*, the local histogram is defined as

(6) $$\begin{array}{c} h_{L}\left(r\right)=\alpha h_{W_{1}}\left(r\right)+\left(1-\alpha \right)h_{W_{2}}\left(r\right). \end{array}$$

Here, *h*_*W*_1__(*r*) is normalized histogram of the neighborhood of center, *h*_*W*_2__(*r*) is normalized histogram of the neighborhood of background, and 0 ≤ *α* ≤ 1.

In summary, steps of CLAHE algorithm are shown as follows.

1. For any point in the image, its relevant region is determined according to the size *W* of window.
2. Compute the histogram of rectangular window according to formula (5) or (6).
3. Carry out equalization for the histogram within the rectangular window, to achieve the processing of the center pixel of the window.
4. Move rectangular window to the next adjacent pixel, and repeat the above process until the whole image is completely processed.

## 4. Experimental Results and Discussion

In this paper, the experiments have been carried out to verify the aforementioned comprehensive image preprocessing methods. Before experiment, we need to take several tests on different parts of DR image so that we can select appropriate values for some specific parameters that include *γ*  factor in gamma correction algorithm, decomposition levels *L* and gain factor *y*_*α*_ in SFL-CT processing algorithm, and *w* and *α*  in CLAHE algorithm.

**Figure 5:**
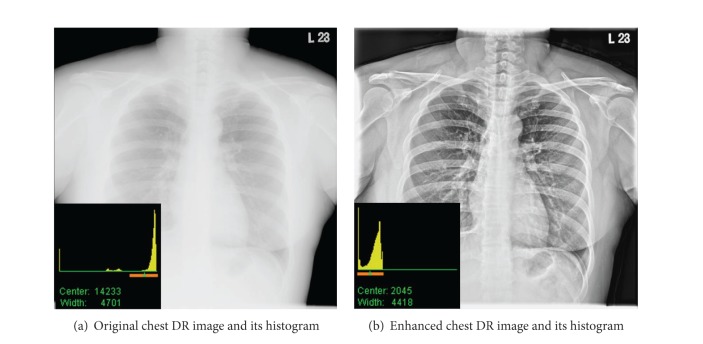
Chest image before and after enhancing.

As shown in Figure 5, the chest images (4096 × 4096 × 16 bits) before and after processing by our proposed methods are totally different. Obviously, the contrast of chest image after preprocessing is greatly enhanced; more details including texture, spine, and limbs can be differentiated; noise and artifacts are suppressed simultaneously. Analyzers can manually select the ROI of histogram with the so-called “window-width” and “window-center” operators to adjust display effect. Window-center operator is to choose the middle value of ROI of histogram, and window-width is to choose the upper and lower limit values of ROI of histogram. For other organs, such as head, spine, abdomen, and limbs, we can also get good image after preprocessing with our proposed algorithm.

In fact, the influence of the various processing algorithms on the flow of image preprocessing to the final results is related to their role sequence; a better visual observation effect can be got only by the flow designed in this paper.

**Figure 6:**
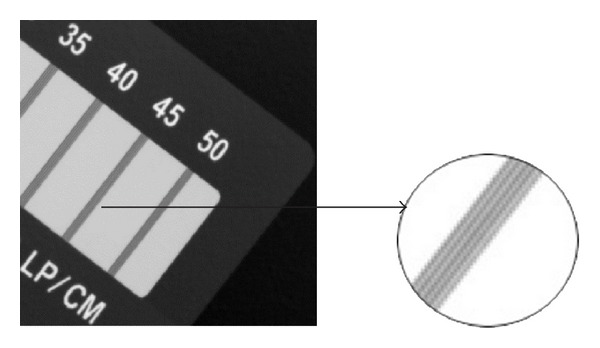
Test image of line-pair card.

Moreover, this CCD-DR imaging system also tests the standard line-pair card.  Figure 6 shows the actual image enhanced by our method. It can be objectively observed that the distinguished line-pair index can reach 4.5 LP/mm (45 LP/CM) which is close to the aforementioned theoretical value, which can prove a higher resolution of imaging.

## 5. Conclusion

Based on a medical X-ray DR imaging system constructed with high-performance CCD camera, this paper proposes a comprehensive preprocessing method which includes flat-field correction, gamma correction, median filter, and a hybrid multiscale contrast enhancement mode. This hybrid enhancement algorithm, which is based on sharp frequency localization-contourlet transform (SFL-CT) and contrast limited adaptive histogram equalization (CLAHE), is verified by the clinical DDR images. Experimental results show that, with appropriate parameters, the proposed comprehensive preprocessing algorithm can greatly enhance the contrast and suppress the artifacts simultaneously.

## Figures and Tables

**Table 1:** Basic parameters of CCD camera.

Effective pixel values	4096 × 4096 = 16.8 million
Image grayscale	65536
Imaging acquisition time	≤5 s
A/D conversion accuracy	16 bit

## References

[1] Zhang M, Mou X (2010). Nonlinear multi-scale contrast enhancement for x-ray chest radiography. *Journal of Xi’an Jiaotong University*.

[2] Vuylsteke P, Schoeters E Multi-scale image contrast amplification (MUSCIA).

[3] Stahl M, Aach T, Dippel S (2000). Digital radiography enhancement by nonlinear multiscale processing. *Medical Physics*.

[4] Do MN, Vetterli M (2005). The contourlet transform: an efficient directional multiresolution image representation. *IEEE Transactions on Image Processing*.

[5] Lu Y, Do MN A new contourlet transform with sharp frequency localization.

[6] Feng P (2012). *Non-Aliasing Multi-Scale Geometric Overlapping Image Analysis Technology and Its Application*.

[7] Feng P, Pan Y, Wei B, Jin W, Mi D (2007). Enhancing retinal image by the contourlet transform. *Pattern Recognition Letters*.

[8] Cromartie R, Pizer SM (1993). Structure-sensitive adaptive contrast enhancement methods and their evaluation. *Image and Vision Computing*.

[9] Louis AJ, Belward J, Altas I (1995). A variational approach to the radiometric enhancement of digital imagery. *IEEE Transactions on Image Processing*.

